# Tailoring polarization and magnetization of absorbing terahertz metamaterials using a cut-wire sandwich structure

**DOI:** 10.3762/bjnano.9.136

**Published:** 2018-05-16

**Authors:** Hadi Teguh Yudistira, Shuo Liu, Tie Jun Cui, Han Zhang

**Affiliations:** 1 SZU-NUS Collaborative Innovation Centre for Optoelectronic Science & Technology and Key Laboratory of Optoelectronic Devices and Systems of Ministry of Education and Guangdong Province, College of Optoelectronic Engineering, Shenzhen University (SZU), Shenzhen 518060, China; 2 Mechanical Engineering Program, Institut Teknologi Sumatera (ITERA), Lampung 35365, Indonesia,; 3 State Key Laboratory of Milimeter Waves, Southeast University, Nanjing 210096, China

**Keywords:** absorber, cut-wire, metamaterial, terahertz

## Abstract

The permittivity and permeability of a cut-wire sandwich structure can be controlled by laterally shifting the upper and lower layers. The use of this process for designing specific application-oriented devices may lack clear-cut guidelines because the lateral misalignment will significantly change the permittivity and permeability simultaneously. Therefore, in this work, we designed, fabricated and characterized a cut-wire sandwich device capable of tailoring the polarization and magnetization separately, thereby providing a promising recipe for achieving specific application objectives, such as a high-performance absorber. Accumulated charges effectively provided the polarization at the edge of cut-wires, and the surface current density on the cut-wires at top and bottom layers effectively generated the magnetization. By controlling and optimizing the geometrical configurations of the entire sandwich device (without lateral misalignment), the impedance could be matched to that of free space while generating a large imaginary part in the refractive index. This work characterizes the absorption performance of such sandwich structures in the terahertz regime. This mechanism could be further extended to other metamaterial devices in the terahertz and other frequency ranges because polarization and magnetization can now be selectively controlled in a straightforward manner.

## Introduction

The terahertz spectrum is located between the infrared and microwave spectrum. This part of the spectrum has unique properties, such as being non-ionizing and subject to considerably less Rayleigh scattering than the visible or infrared spectrum [1]. The terahertz spectrum has been widely used in research fields such as medical imaging [1–3] and security applications [4].

Optical [5–7] and microwave metamaterials [8–10] have been intensively investigated in the past decades. Although detecting the terahertz wavelengths is somewhat difficult, through the development of terahertz detection technology, the study of terahertz metamaterials has been reported as early as 2004 [11], which is earlier than the study of optical metamaterials.

The materials consist of multiple tiny metallic structures fabricated on dielectric substrates for metamaterial applications. The length of the tiny metallic structures of terahertz metamaterials is usually approximately hundreds of micrometers [12–14], which is much smaller than the wavelength of the terahertz electromagnetic (EM) wave. Thus, such structures can be considered a homogeneous medium from the perspective of the EM wave. The existence of multiple metallic structures in a device may affect the EM properties, such as permittivity and permeability.

Several works on metamaterial absorbers have been presented such as split-ring resonators [15], electric-field-coupled (ELC) resonators [16], lossy cut-wire bars [17], and donut-type resonators [18]. Most previous works on the perfect absorber have been explained by interference theory [19–23], for example that presented by Chen [19] who described a perfect metamaterial absorber based on the interference theory. He demonstrated numerical simulations and analytical calculations of the metamaterial absorber. The design of the metamaterial absorber comprised two parts: a metallic plane, which serves as the background, and the metamaterial structure. The two parts are separated by a dielectric spacer. Another necessary condition found was that absorbers can be achieved by increasing the imaginary part of the refractive index and matching the impedance of the metamaterial with air impedance [24–25].

Cut-wire and split-ring structures are often used as electric resonators that can control the permittivity of a metamaterial [26–28]. By using a cut-wire sandwich structure, the permeability can be controlled due to the existence of a looping surface current between the cut-wire structures. The cut-wire structure is sensitive to polarization. Symmetric geometries are required to produce a metamaterial with arbitrary polarization. The cross-shaped structure is one example of a symmetric geometry that is based on the cut-wire structure and has been used to design terahertz absorbers [29]. The star-shaped structure is another structure with a symmetric geometry that is based on the cut-wire structure. One of the unique features of the star-shaped structure is its capability to exhibit more than one resonance peak [30].

Presently, the permittivity and permeability of the cut-wire sandwich structure can be adjusted by laterally shifting the upper and lower layers of the cut-wire structure [27,31–32]. The symmetric breaking in cut-wire sandwich structures can simultaneously generate negative values for permittivity and permeability [27,32], thereby making a perfect lens [33]. The cut-wire sandwich structure can be organized to achieve another specific application objective, that is, a high-performance absorber. By controlling and optimizing the geometrical configurations of the entire sandwich device without lateral misalignment, the impedance can be matched to that of free space while generating a large imaginary part in the refractive index.

In this work, we theoretically and experimentally studied the absorbance of a thin terahertz metamaterial that was based on the cut-wire sandwich structure, which was demonstrated to generate polarization and magnetization simultaneously. By using cut-wire sandwich structures, we could control the permittivity and permeability of the metamaterial to match its impedance to that of free space and realize a high imaginary refractive index value. Simple cut-wire, cross-shaped and star-shaped sandwich structures, composed of one, two, and four metallic cut-wire bars, respectively, are presented in this paper. By arranging the cut-wire structure on a unit cell, we could obtain a metamaterial absorber with a specific polarization response (arbitrary response or sensitive response) and a specific resonance feature.

## Experimental

**Figure 1:**
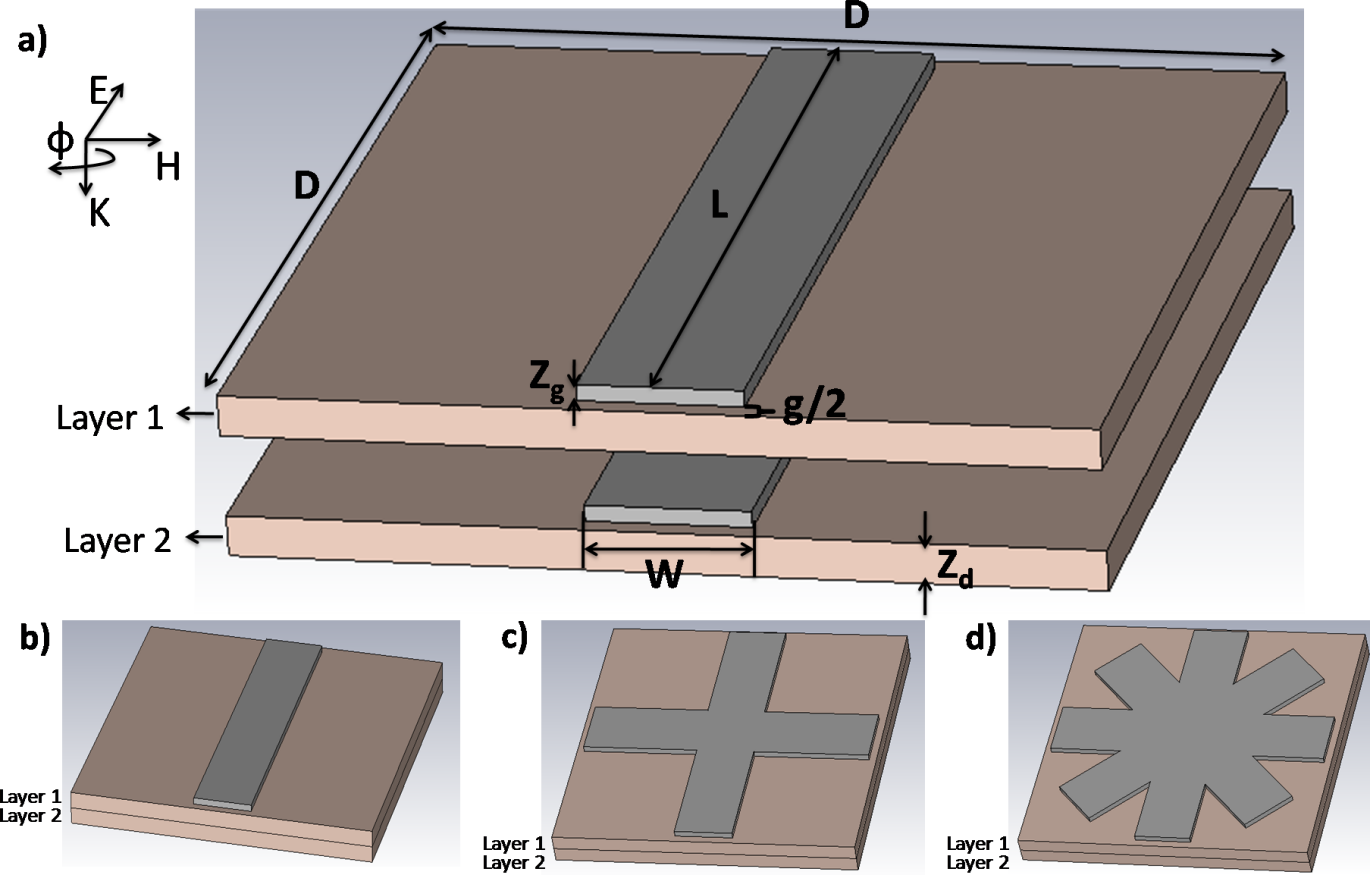
a) Illustration of cut-wire sandwich structure. *W*, *L*, *g*, *Z*_d_ and *Z*_g_ are width of cut wire (20 µm), length of cut wire (100 µm), the gap size of cut wire with its neighbor (5 µm), polyimide substrate thickness (5 µm), and tge cut-wire structure thickness (100 nm), respectively. *D* is the lattice constant (*D* = *L* + *g*). b) Sample 1: cut-wire sandwich structure, c) Sample 2: cross-shaped sandwich structure, d) Sample 3: star-shaped sandwich structure.

Figure 1a illustrates the geometry of the cut-wire sandwich structure. The width of the gold metallic bar (*W*), the length of gold metallic bar (*L*), the gap size between two unit cells (*g*), the thickness of the gold metallic bar (*Z*_g_), the thickness of the dielectric (polyimide (PI)) (*Z*_d_), and the lattice constant (*D* = *L* + *g*) are 20 μm, 100 μm, 5 μm, 100 nm, 5 μm and 105 μm, respectively. Gold was selected for the metallic bars to ensure good conductivity and avoid oxidation in air. We fabricated three samples: cut-wire (Figure 1b), cross-shaped (Figure 1c) and star-shaped sandwich structures (Figure 1d). 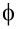 is the angle that described the EM polarization direction. The cross-shaped sandwich structure was composed of two cut-wires that were arranged perpendicular to each other. The star-shaped sandwich structure was composed of four cut-wires that were arranged every 45° of 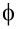.

The impedance (*z*) and refractive index (*n*) are the two main parameters that describe the EM properties of a material and can be defined as 
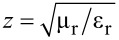
 and 
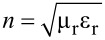
, respectively, where ε_r_ and μ_r_ are the relative permittivity, and relative permeability, respectively. The relative permittivity of the material can be defined as ε_r_ = 1 + (*P* / ε_r_*E* ), and the relative permeability can be defined as μ_r_ = 1 + *M* / *H*, where *E*, *H*, *P*, and *M* are the electric field, magnetic field, polarization, and magnetization [12], respectively. Magnetization and polarization are two factors that can be used to tailor the relative permeability and relative permittivity, respectively. The important parameters of a cut-wire sandwich structure are the gap size between two unit cells and the thickness of the dielectric spacer. Accumulation charge is located at the edge of the arm and generates a very large electric dipole moment, which then leads to a large effective permittivity. The effective permittivity increases with a decrease in gap width [12]. The anti-parallel surface current density on the gold electrode line at layer 1 and 2 effectively generate magnetization, which results in a large effective permeability. Both cut-wire structures were separated by a thin polyimide (PI) layer. To match the impedance of the metamaterial to that of free space, the effective permittivity should be equal to the effective permeability at the desired frequencies.

**Figure 2:**
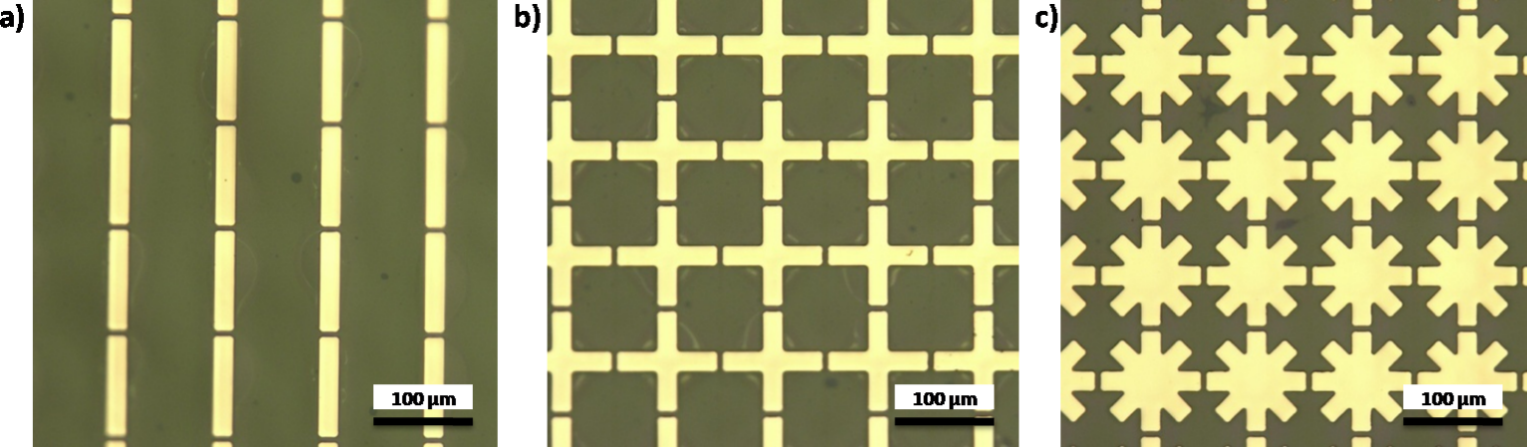
Microscopy images of the fabrication samples: a) Cut-wire sandwich structure, b) cross-shaped sandwich structure, and c) star-shaped sandwich structure.

Samples with area of 10 × 10 mm were fabricated. First, a PI layer (Yi Dun New Materials, Suzhou Co., Ltd.) was spin-coated on a silicon wafer and then baked on a hot plate at 80 °C, 120 °C, 180 °C and 250 °C for 5, 5, 5 and 20 min, respectively. Then, standard photolithography was performed [34–35], and another Ti/Au layer (30/100 nm) was deposited onto the PI substrate by electron beam evaporation. A standard lift-off process was employed to enable the formation of the final metallic pattern. The second layer was fabricated by repeating the above processes. The final process involved peeling off the cured PI layer from the silicon wafer. Figure 2 exhibits microscopy images of the fabricated samples.

## Results and Discussion

The absorbance (*A*(ω)) of the material was calculated from the reflectance (*R*(ω)) and transmittance (*T*(ω)) by using the following equation: *A*(ω) = 1 − *R*(ω) − *T*(ω). Smith et al. [36] defined transmittance and reflectance as

[1]
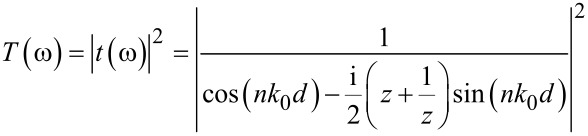


and

[2]
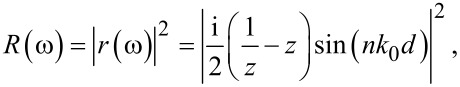


Where *t*(ω), *r*(ω), *n*, *k*_0_, *d* and *z* are the transmissivity, reflectivity, refractive index, wave number in free space, substrate thickness and impedance, respectively. In the matching air impedance condition (i.e., *z* = 1 + i0), the reflectance would be zero and Equation 1 is expressed as

[3]
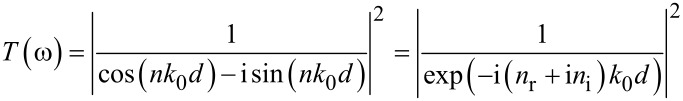


where *n*_r_ and *n*_i_ are the real and imaginary parts of the refractive index, respectively. Equation 3 can be simplified as

[4]
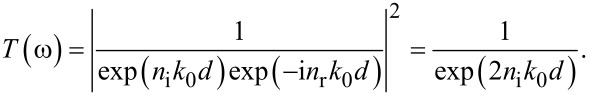


Equation 4 shows that a high imaginary refractive index is required to achieve zero transmittance. To achieve high absorbance, the impedance of the metamaterial should match the air impedance and the imaginary part of the refractive index value should be high.

**Figure 3:**
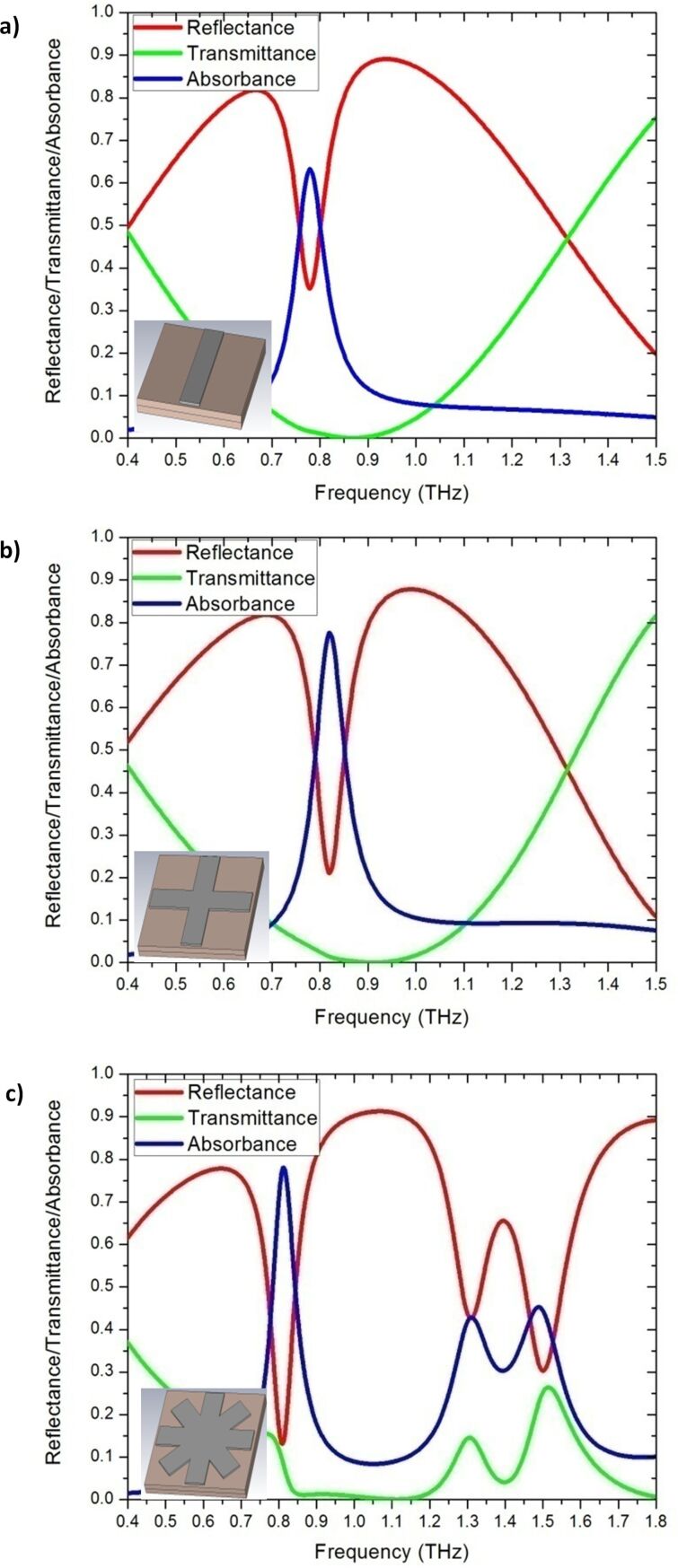
Reflectance–transmittance–absorbance (RTA) simulation results: a) Cut-wire sandwich structure, b) cross-shaped sandwich structure, and c) star-shaped sandwich structure.

**Figure 4:**
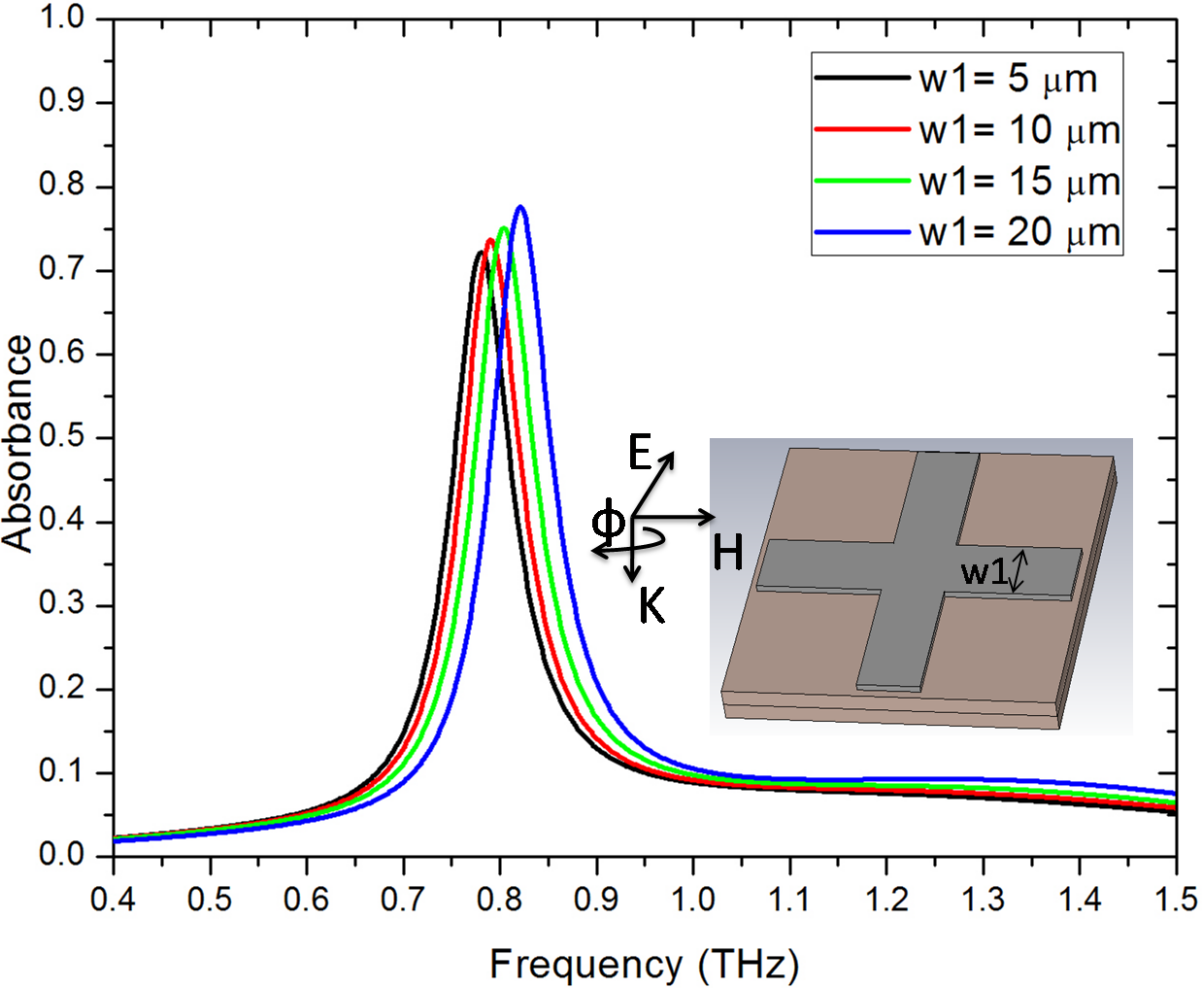
The simulation result of the absorbance difference for the cut-wire width of the cross-shaped sandwich structure at 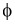 = 90° with w1 varying from 5 µm to 20 µm.

The electrical properties of the thin metallic bar deposited on the substrate strongly deviated from that of the bulk metallic [37]. The Drude model [38] was used for calculating the permittivity of thin gold metallic bars in the software CST microwave studio [39], where


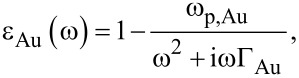


with ω_p,Au_ = 1.38 × 10^16^ rad/s and Γ_Au_ = 0.11 × 10^15^ s^−1^. ω, ω_p,Au_ and Γ_Au_ are the angular frequency, angular plasma frequency of gold, and damping constant of gold, respectively. Figure 3 presents the reflectance–transmittance–absorbance (RTA) simulation result of cut-wire (Figure 3a), cross-shaped (Figure 3b) and star-shaped sandwich structures (Figure 3c). The simulation results of the cut-wire sandwich structure exhibited a narrow absorbance of 63% at 0.78 THz. The absorbance of the cross-shaped sandwich structure reached 78% at 0.82 THz. The full width at half maximum absorbance of the cut-wire sandwich structure and that of the cross-shaped sandwich structure are 0.05 and 0.06 THz, respectively. The narrow absorbance of these structures may due to the conductivity of the thin metallic bar. The frequency peak of the absorbance of the cut-wire sandwich structure differed from that of the cross-shaped sandwich structure, and this discrepancy could be explained as follows. The existence of the cut-wire width of the cross-shaped sandwich structure at 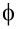 = 90° reduced the metallic bar length parallel to the external electric field by a few micrometers, thereby, increasing the resonance frequency. Figure 4 shows the simulation result of the absorbance difference as the cut-wire width (w1) of the cross-shaped sandwich structure at 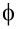 = 90° was varied from 5 µm to 20 µm. The frequency peak of the absorbance of the cross-shaped sandwich structure decreased to near that of the cut-wire sandwich structure when the cut-wire width of the cross-shaped sandwich structure at 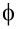 = 90° was decreased. This change in cut-wire width of the cross-shaped sandwich structure at 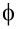 = 90° resulted in an alteration of the polarization and magnetization on the unit cell and thus altered the absorbance magnitude and location of the frequency peak. The surface current flowed only on the cut wire that was parallel to the external electric field. Meanwhile, no surface current flowed along on the cut wire at 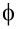 = 90° on the cross-shaped sandwich structure. Consequently, polarization and magnetization were generated only on the cut wire that was parallel to the external electric field. The star-shaped sandwich structure had three absorbance peaks: 78% at 0.81 THz, 43% at 1.31 THz and 45% at 1.5 THz. The full width at half maximum absorbance of the star-shaped sandwich structure at 0.81 THz was 0.06 THz, which is close to that of the cut-wire sandwich structure. The direction of the surface current flow was parallel to the external electric field at 0.81 THz on the star-shaped sandwich structure, and this pattern was similar to that of the cross-shaped structure. At 1.31 THz, the surface current flow was along the cut wire not only at 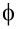 = 0°, but also at 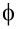 = 45°. Magnetization was generated on the cut wire at 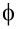 = 45° at 1.33 THz because an anti-parallel surface current existed on both cut-wires at 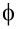 = 45°. The surface current flow direction at 1.5 THz was opposite to that at 1.31 THz.

The scattering parameters (S-parameters) were used to extract the EM properties of the proposed absorber [36,40]. The refractive index and impedance were obtained using Equation 5 and Equation 6 [40] as

[5]
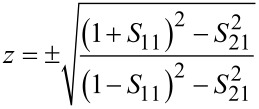


**Figure 5:**
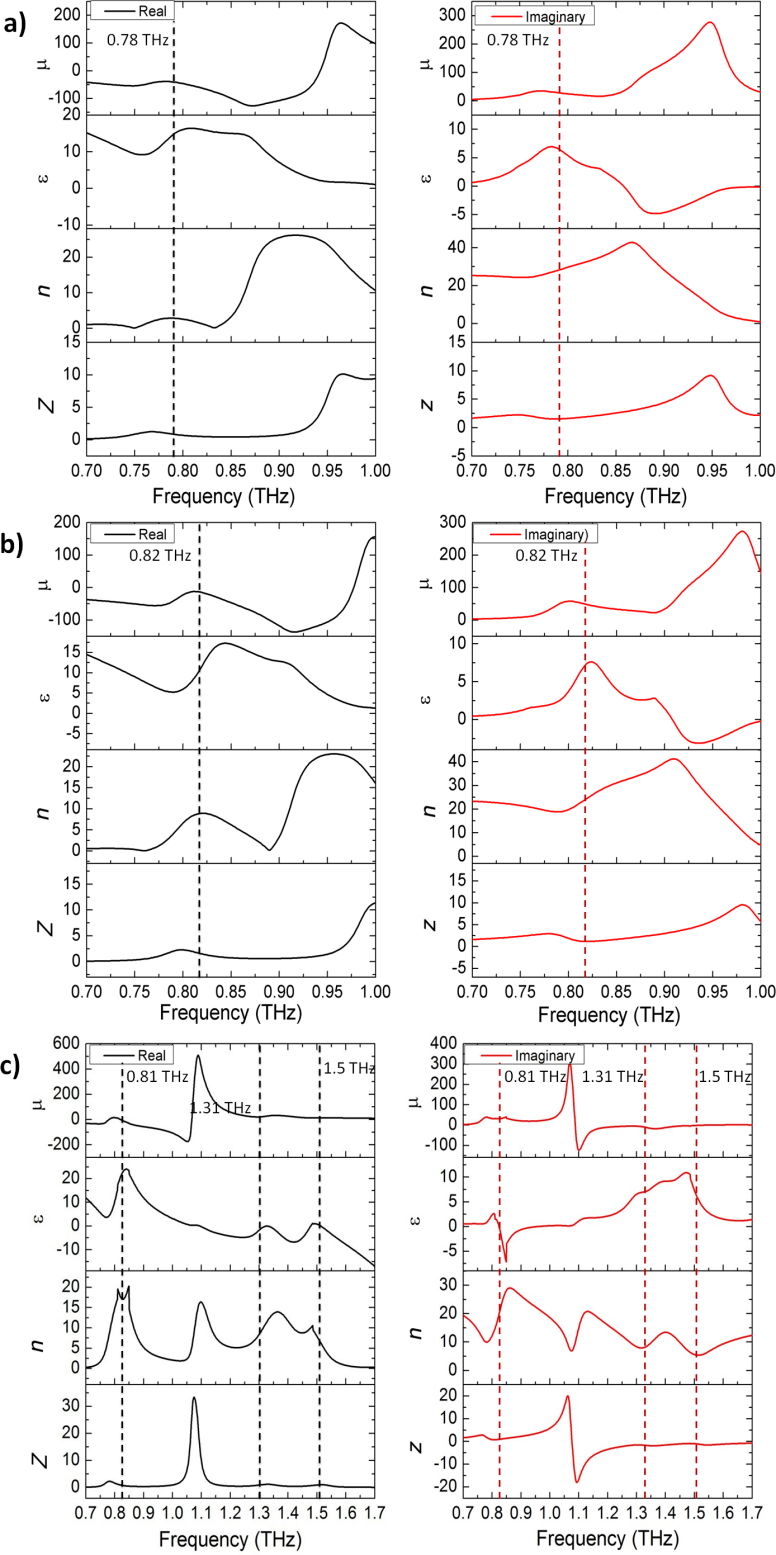
Permeability (µ), permittivity (ε), refractive index (*n*), and impedance (*Z*) properties of: a) Cut-wire sandwich structure, b) cross-shaped sandwich structure, and c) star-shaped sandwich structure. The black and red curves represent the real and imaginary part of each value, respectively.

[6]
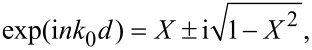


where *X* = (1 / 2*S*_21_^2^)(1 – *S*_11_^2^ + *S*_21_^2^). The metamaterial was considered a passive medium; hence, the sign in Equation 5 and Equation 6 was determined according to *z*_r_ ≥ 0 and *n*_i_ ≥ 0, where *z*_r_ is the real part of the impedance. The refractive index and impedance are related to permittivity and permeability by the relations ε = *n* / *z* and μ = *nz* [36,40]. Figure 5a–c presents the EM properties of the cut-wire, cross-shaped and star-shaped sandwich structures, respectively. The narrow band absorbance was obtained by matching the impedance of the absorber with the impedance of air, thus realizing a high imaginary part of the refractive index at absorption frequencies. To achieve an impedance that was close to that of air, the permeability (µ) value should approach the permittivity (ε) value. The black and red curves in Figure 5 represent the real and imaginary values, respectively. Figure 5a shows that the µ (real), ε (real), µ (imaginary) and ε (imaginary) parts of the cut-wire sandwich structure at 0.78 THz were −38.62, 12.4, 33.13 and 6.88, respectively. The *Z* value of the cut-wire sandwich structure at 0.78 THz was 1.08 + i1.55, which was close to the impedance of air, i.e., 1 + i0. The refractive index (*n*) of the cut-wire sandwich structure at 0.78 THz was 2.71 + i26.72. The imaginary part of the refractive index of the cut-wire sandwich structure was high and could thus yield low transmittance. The *Z*-value of cross-shaped structure at 0.82 THz and the star-shaped sandwich structure at 0.81 THz were 1.48 + i1.15 and 1.18 + i0.75, respectively, which were also near the air impedance value and thus could yield a low reflectance. The absolute *Z*-values of the cut-wire sandwich structure at 0.78 THz, the cross-shaped sandwich structure at 0.82 THz and the star-shaped sandwich structure at 0.81 THz were 1.89, 1.87 and 1.4, respectively. The absolute *Z*-values of the star-shaped sandwich structure were the closest to the absolute air impedance value of 1, and thus yielded the lowest reflectance of all samples. The reflectance of the cut-wire sandwich structure at 0.78 THz was slightly higher than that of the cross-shaped sandwich structure at 0.82 THz because the absolute *Z*-value of the cross-shaped structure was closer to the absolute air impedance value as compared to that of the cut-wire sandwich structure. The *n*-values of the cross-shaped structure at 0.82 THz and the star-shaped sandwich structures at 0.81 THz were 8.94 + i24.79 and 19.59 + i14.96, respectively. The imaginary part of the refractive index of the star-shaped sandwich structure at 0.81 THz is lower than that of the cut-wire sandwich structure at 0.78 THz and the cross-shaped sandwich structure at 0.82 THz. The transmittance of the star-shaped sandwich structure at 0.81 THz could not reach zero due to the small value of the imaginary part of the refractive index. The *Z*-values of the star-shaped sandwich structure at 1.31 THz and 1.5 THz were 1.01 − i1.51 and 0.87 − i0.98, respectively. These values were close to the impedance of air, and therefore, were able to achieve a low reflectance. The imaginary parts of the refractive index of the star-shaped sandwich structure at 1.31 THz and 1.5 THz are 7.99 and 5.64, respectively. These figures were much lower than the imaginary part of refractive index of the star-shaped sandwich structure at 0.82 THz. Therefore, the transmittance of the star-shaped sandwich structure at 1.31 THz and 1.5 THz was higher than that of the star-shaped sandwich structure at 0.82 THz.

**Figure 6:**
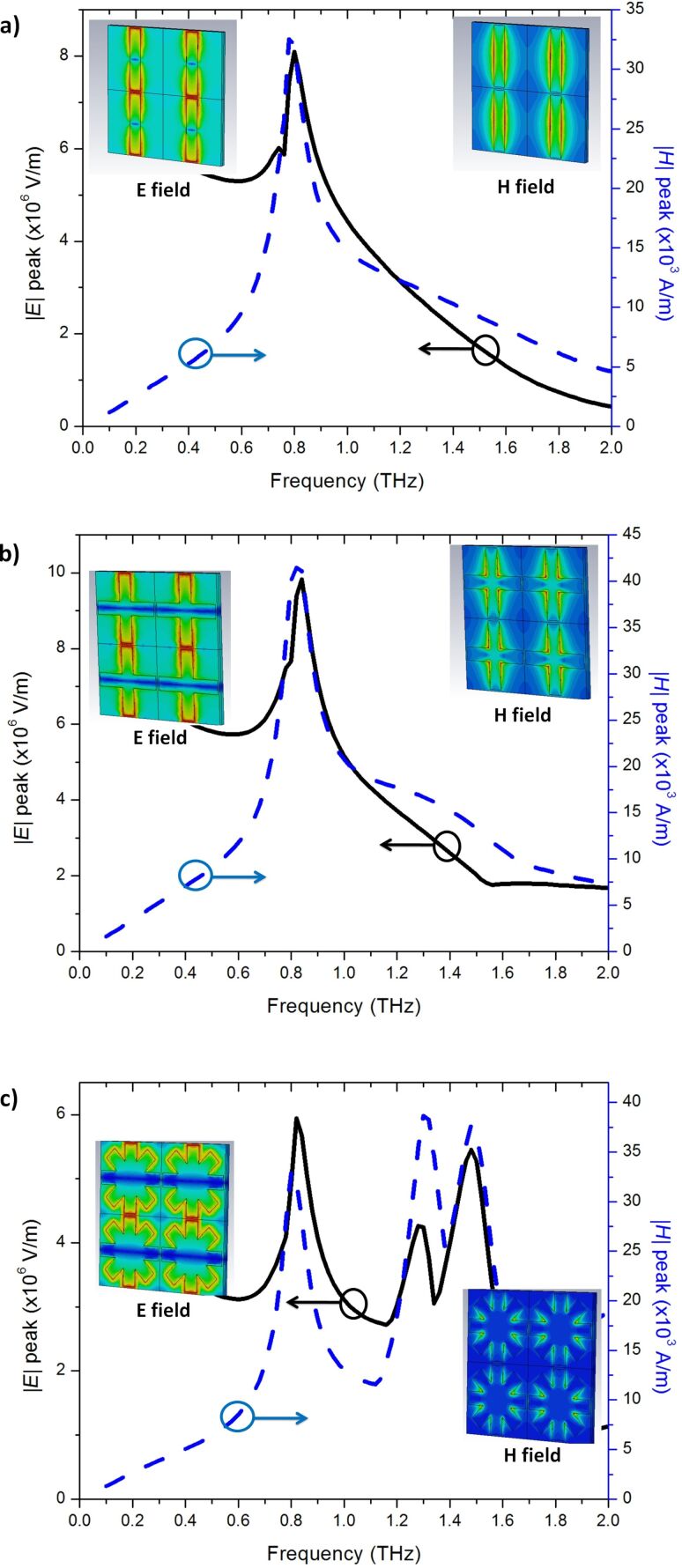
The peak value of electric field and magnetic field of: a) Cut-wire sandwich structure, b) cross-shaped sandwich structure, and c) star-shaped sandwich structure. The insets of (a–c) in each panel show electric and magnetic field distributions of the cut-wire sandwich structure at 0.78 THz, cross-shaped sandwich structure at 0.82 THz, and star-shaped sandwich structure at 0.81 THz, respectively.

**Figure 7:**
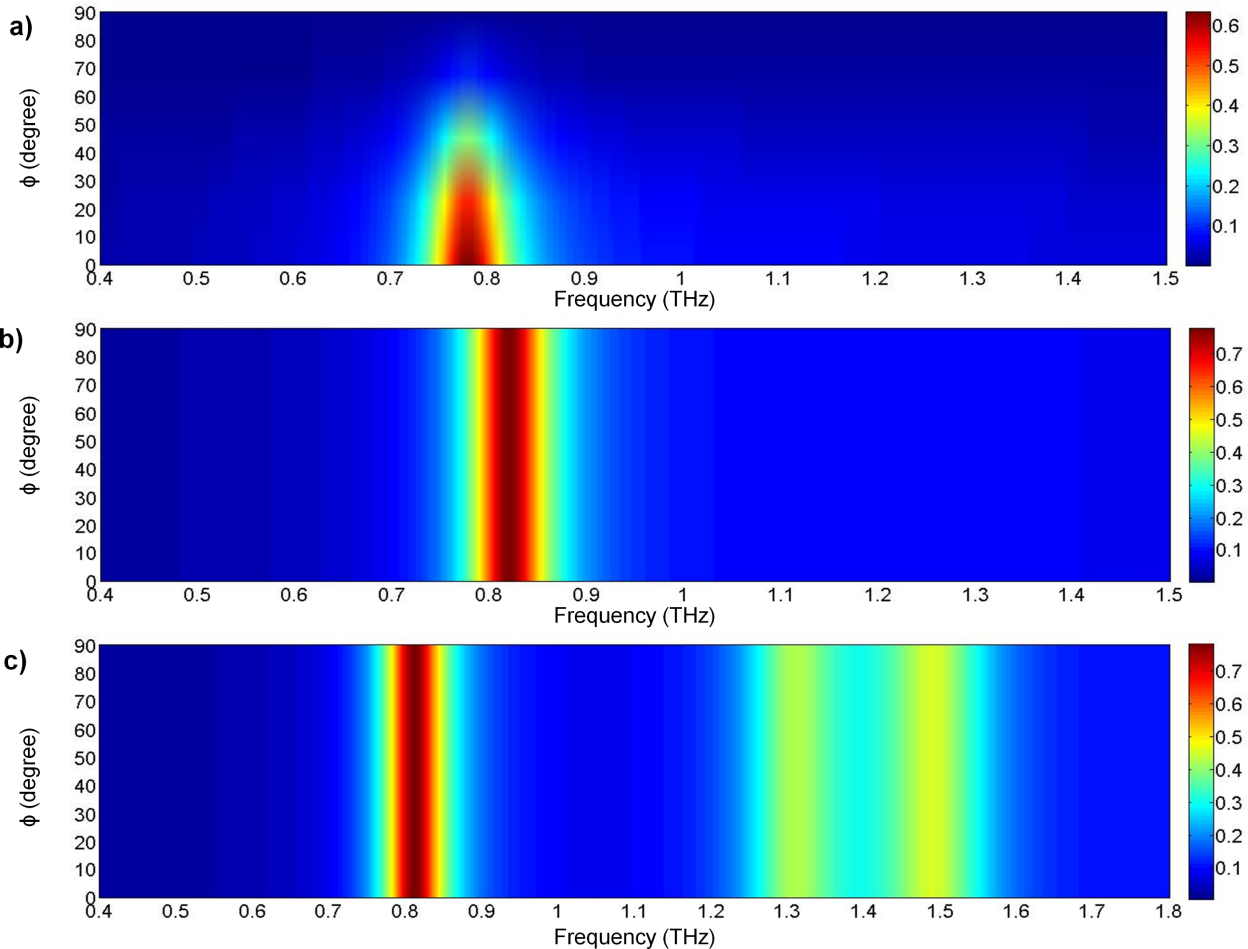
The simulation result of the absorbance difference for polarization angles from 0° to 90° of: a) Cut-wire sandwich structure, b) cross-shaped sandwich structure, and c) star-shaped sandwich structure.

Figure 6a–c presents the peak magnitude of the electric and magnetic fields of the cut-wire, cross-shaped and star-shaped sandwich structures, respectively. The frequencies of the peak values of the electric and magnetic fields were similar to those of the absorbance peaks. This condition indicated the existence of a high absorption when the high electric field and magnetic field are stored on the metamaterial structure. The electric and magnetic field distributions are presented in the inset of Figure 6. The electric field distribution showed that the maximum value of the electric field was at the gap between two unit cells. The magnetic field distribution showed that the maximum value of the magnetic field was between layers 1 and 2. The anti-parallel surface in the gold metallic bars on layers 1 and 2 generated magnetization. The maximum value of the electric and magnetic fields was found here, while the *Z*-values of the samples were close to the impedance of air and the imaginary value of the refractive index was high at a similar frequency (Figure 5). Figure 7a–c presents the simulation results of absorbance with different polarization directions for the cut-wire, cross-shaped and star-shaped sandwich structures, respectively. The cut-wire sandwich structure was demonstrated to be a metamaterial with sensitive polarization. Figure 7a shows that the absorbance of the cut-wire sandwich structure was zero when the external electric field was rotated by 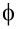 = 90°. The EM wave was fully transmitted through the cut-wire sandwich structure when the external electric field is rotated by 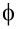 = 90° because polarization and magnetization were not generated. Meanwhile, the cross-shaped and star-shaped sandwich structures presented metamaterials with arbitrary polarization. The absorbance of these structures presented an identical performance when the external electric field was arbitrarily polarized.

**Figure 8:**
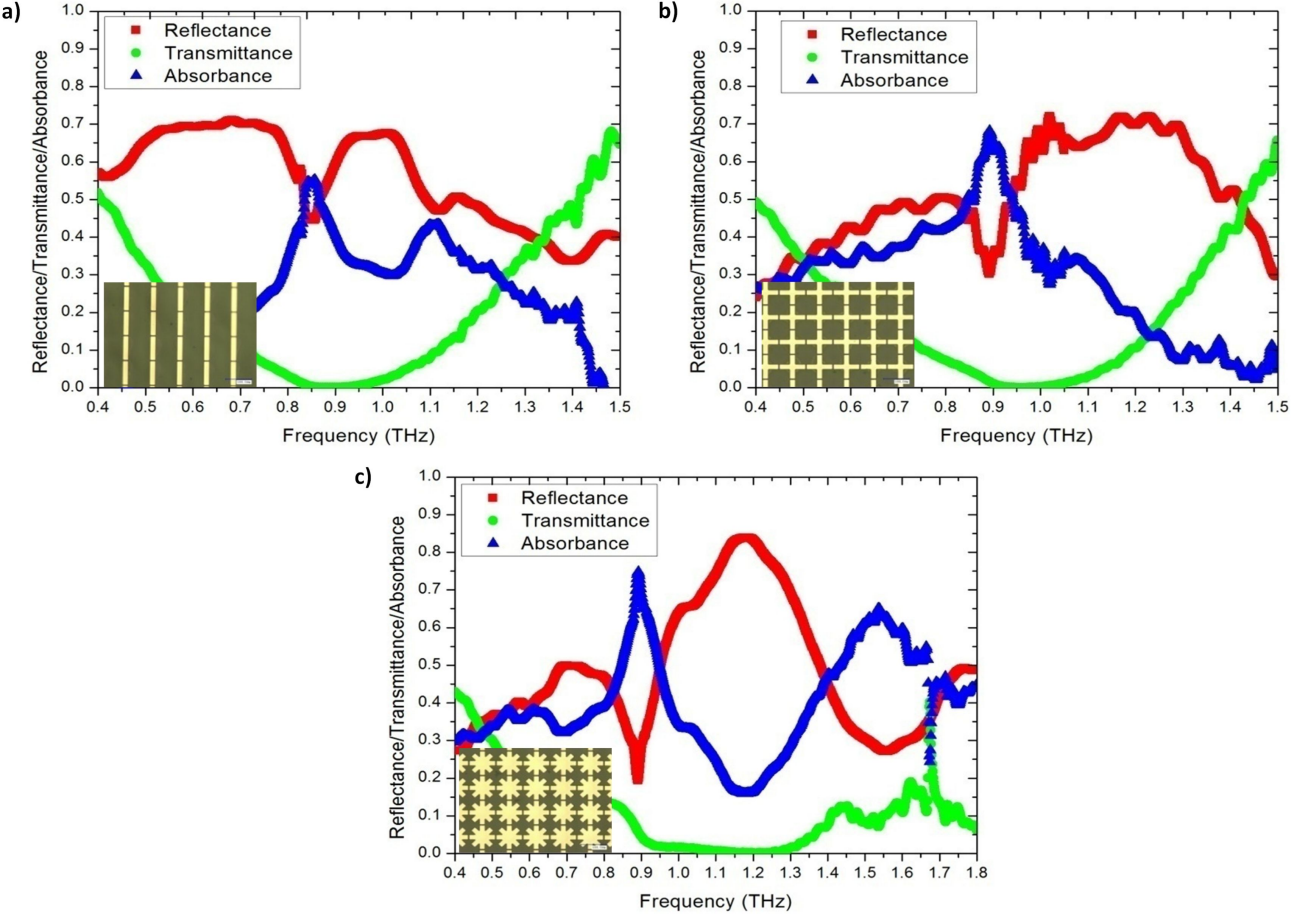
The measured results of reflectance, transmittance and absorbance under normal incidence: a) Cut-wire sandwich structure, b) cross-shaped sandwich structure, and c) star-shaped sandwich structure.

**Figure 9:**
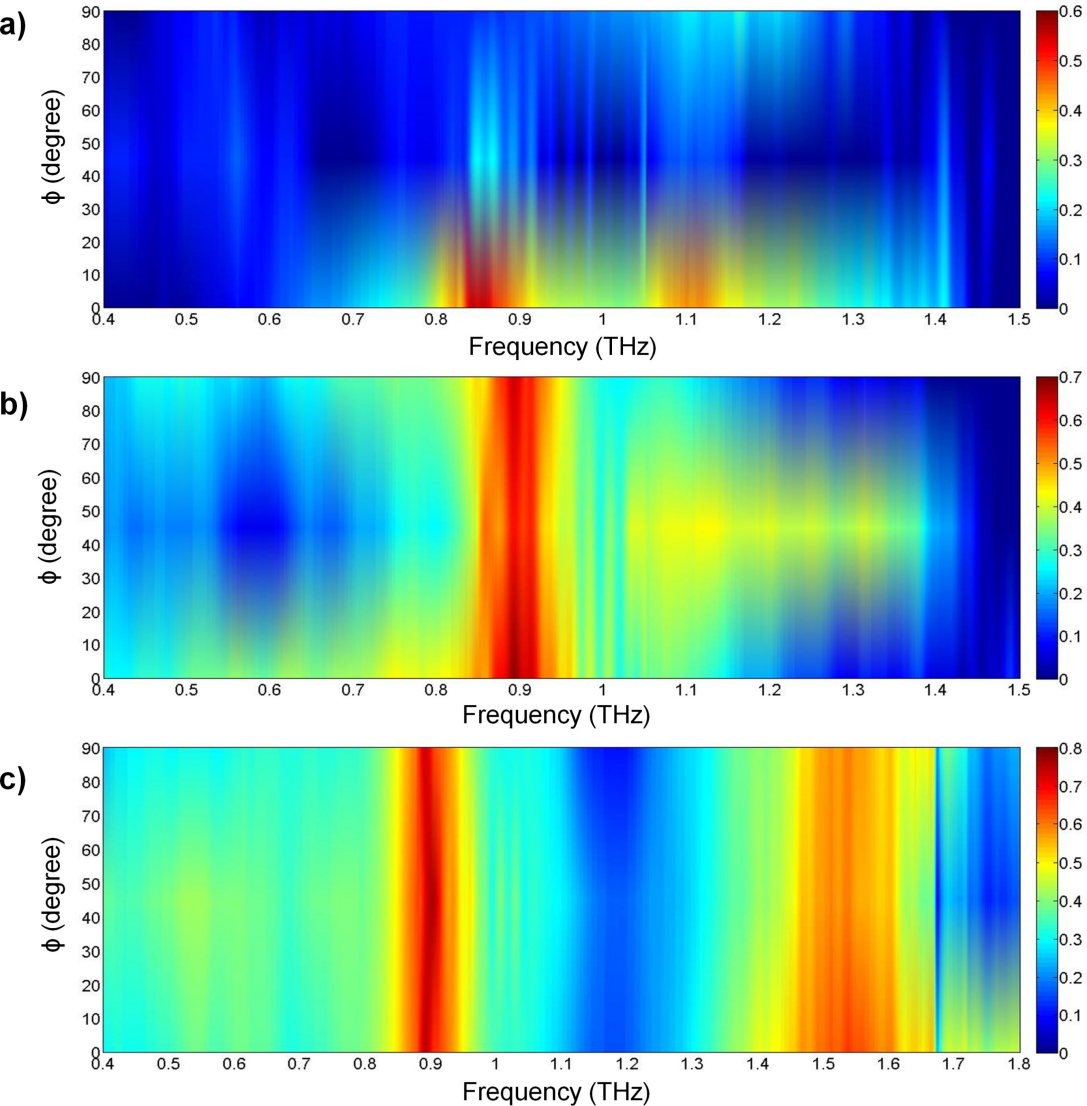
Measured absorbance results for polarization angles from 0° to 90° for: a) Cut-wire sandwich structure, b) cross-shaped sandwich structure, and c) star-shaped sandwich structure.

A commercial terahertz time domain spectrometer (Zomega-Z3), whose available spectrum range was from 0.3 THz to 3.0 THz, was applied to characterize the transmission and reflection of the designed absorber. All measurements were conducted in a nitrogen environment (humidity less than 1%) at 25 °C. We first recorded the waveform reflected from an opaque gold film as reference for the reflection. For the transmission reference, we recorded the waveform transmitted without any sample. Figure 8a–c presents the measured RTA results of the cut-wire, cross-shaped and star-shaped sandwich structures, respectively. The measured absorbance reached the peak value of 55% at 0.85 THz for the cut-wire sandwich structure, 68% at 0.89 THz for the cross-shaped sandwich structure, and 75% at 0.89 THz for the star-shaped sandwich structure. The reduction of the measured resonance frequencies and the slightly reduced absorbance measurement results in all of the samples were due to fabrication inaccuracies. The corner of the metallic bar structure was slightly rounded (Figure 2) and the PI thickness were slightly different than anticipated. The EM properties of the samples were also altered due to inaccuracies in the fabrication process. Landy et al. reported similar phenomena [25]. The authors fabricated an absorber composed of a two-layer metamaterial. A cross-shaped structure and an electrically coupled ring resonator (ERR) were separated by a layer of benzocyclobutane (BCB) as a dielectric substrate. The sample was fabricated on a high-resistivity thick silicon substrate with 1 mm thickness. Their simulation results showed that a peak absorptivity of 95% at 1.13 THz was reached and the fabricated structure reached a measured absorptivity of 65% at 1.145 THz. Moreover, the authors found that the metallic bar in the fabrication structure was slightly rounded and the thickness of the BCB layer deviated from that predicted. Figure 9a–c presents the measured absorbance with different polarization directions for the cut-wire, cross-shaped and star-shaped sandwich structures, respectively. Figure 9 shows good agreement with the simulation results shown in Figure 7.

## Conclusion

We theoretically and experimentally presented a thin terahertz metamaterial absorber that is based on the cut-wire sandwich structure. The gap between two unit cells and the anti-parallel surface current on the metallic bars yielded polarization and magnetization, respectively. The cut-wire sandwich structure was shown to be a metamaterial with sensitive polarization, whereas the cross-shaped and star-shaped sandwich structures were found to be metamaterials with arbitrary polarization. The star-shaped sandwich structure generated triple-peak absorbance in the simulation. However, the measured results showed a dual-peak absorbance spectrum. A high absorbance performance was obtained in the simulation. The slightly reduced measurement results were mainly due to inaccuracies in the fabrication process. Overall, the trend of the measured absorbance agreed well with the trend of the simulated absorbance.
